# Small Bait Traps May Not Accurately Reflect the Composition of Necrophagous Diptera Associated to Remains

**DOI:** 10.3390/insects12030261

**Published:** 2021-03-20

**Authors:** Kathleen LeBlanc, Denis R. Boudreau, Gaétan Moreau

**Affiliations:** Département de Biologie, Université de Moncton, Moncton, NB E1A 3E9, Canada; ekl4765@umoncton.ca (K.L.); edb5864@umoncton.ca (D.R.B.)

**Keywords:** animal carcasses, bait attraction, decaying substrate, insect succession, forensic entomology, postmortem interval, vertebrate decomposition

## Abstract

**Simple Summary:**

Fly families such as Calliphoridae and Muscidae contribute to the decomposition of cadavers and play an important role in courtroom proceedings, in part because of the clues they provide to help determine the time of death. In forensic entomology studies, they are often sampled using small bait traps containing a small amount of decomposing animal tissue. To determine whether the fly assemblages recovered by small bait traps are similar to those found on whole remains, we simultaneously documented the flies found on domestic pig carcasses and within small traps baited with pork liver. Results indicated that the fly assemblages found in the small bait traps and on the carcasses were different and reinforced the fact that caution should be exercised when data obtained from small bait traps are used in court.

**Abstract:**

Small bait traps are beginning to emerge in forensic entomology as a new approach to sample early-colonizing necrophagous Diptera species while reducing the investment in time and energy in obtaining information. To test the hypothesis conveyed by the literature that these traps can be a substitute for whole carcasses, we simultaneously documented the Diptera assemblages visiting and colonizing domestic pig carcasses and small traps baited with pork liver. Results indicated that Diptera species occurrence and assemblage composition in the small bait traps and on the carcasses differed, while they were similar when comparing only the pig carcasses. These results are in agreement with the literature that examined insect colonization of other decaying substrates. Although small bait traps can be useful tools to document the communities of necrophagous Diptera in a given area, we stress that caution must be exercised when extending the data obtained by these traps to courtroom proceedings.

## 1. Introduction

Passive data retrieval methods are widely used in different fields of research to reduce the investment in time and energy in obtaining information [1,2,3,4]. This trend is for example present in the field of forensic entomology, which is the study of the association between insects and corpses for forensic and legal purposes, often to determine the post-mortem interval (PMI) of a corpse [5,6,7]. In this field of applied research, insect traps are increasingly used for the sampling of necrophagous Diptera [1,8], one of the key sarcosaprophagous faunal groups [5,9]. Different models of large and small bait traps have been developed, the more commonly used traps in forensic entomology being bottle traps, Schoenly traps, sticky target traps, wind-oriented traps, funnel traps, and bait bins [1,3,8,9,10,11,12,13]. Schoenly traps are baited with whole carcasses [3]. They have proven to be more representative of the colonization of carcasses by both Diptera and Coleoptera than traditional forensic methods. The latter involves direct manual capture using tools such as sweep nets, brushes, tweezers, and aspirators [9]. However, Schoenly traps represent a significative investment and their deployment in a large-scale entomological monitoring program or in a highly urbanized environment might be unrealistic.

Small bait traps are often advantageous compared to the human cadavers, vertebrate carcasses, or Schoenly traps because they emit a faint odor and there are no ethical, legal, or convenience implications associated to their use [1]. These traps mainly target Diptera, notably blowflies (Diptera: Calliphoridae), which are often the first arthropods to colonize carcasses [14]. They are generally baited with a small amount, being 50–200 g, of various animal organs such as pork muscle, pork liver, beef liver, chicken liver, or squid [1,12,15,16]. Synthetic compounds such as sodium sulfide, which act as constant chemical attractants to necrophagous Diptera, may also be used as bait, often with pork liver [8,11,13,17,18]. These small bait traps are emerging in forensic entomology as a method to record the local biodiversity of early-colonizing necrophagous Diptera species [1,15,19], which is known to vary according to the environmental conditions and the geographical region (e.g., [1,5,9]). The literature herein indeed shows that small bait traps are increasingly used to develop databases by forensic institutes and forensic medicine departments in various countries such as Australia, Austria, Brazil, Canada, Croatia, Germany, Portugal, Romania, Spain, Turkey, the United Kingdom, and the United States, to name a few. However, to our knowledge, no experiment has been done to statistically compare the composition of the insect assemblages found in these traps and on whole carcasses placed simultaneously in the same habitat. Nevertheless, some authors have claimed that small bait traps can be a replacement for human cadavers, based on the untested assumption that the bait must emit volatile organic compounds similar to those emitted by a cadaver/carcass in the early stages of decomposition [1,20]. This claim seems to be at odds with the literature indicating that the amount of animal carrion available has a significant impact on its visitation and colonization by Diptera in terms of their abundance and composition (e.g., [5,15,21,22]). As two very different animal substrates, sampling approaches and sampling intervals are compared, we have difficulty believing that previous studies have been able to obtain similar insect assemblages, even in the early stages of decomposition.

To test the claim that small bait traps can be a substitute for whole cadavers or carcasses, which affects the interpretation of data from these traps in case scenarios, we examined the visitation by necrophagous Diptera of domestic pig carcasses (*Sus scrofa domesticus*) and commercial traps baited with 60 g of pork liver. Domestic pig carcasses of ~22 kg are considered the best replacement for human cadavers, due to their anatomical characteristics, diet, replicability and practicality, and fur [10,23,24]. The hypothesis tested here is that the attractiveness of carrion to Diptera changes with species interactions, decomposition, and amount of available tissue. From this and the above cited literature, we predict that the Diptera assemblage collected on carcasses and in small bait traps will differ. If this prediction is rejected, we predict that the trapped insects will be representative of those which are collected on remains for a short period in early decomposition. Nevertheless, we not only documented early decomposition but prolonged the study until the dry stage to determine whether the small bait traps may be representative of a later decomposition stage, because they were baited with partially decomposed internal tissues.

## 2. Materials and Methods

### 2.1. Experimental Design

Domestic pig carcasses weighing 22.6 ± 3.3 kg were purchased from a certified hog farm located approximately 60 km from the study site. The animals were killed using a compressed air pistol with a retractable pin placed directly on the forehead. They were immediately placed in two airtight plastic bags to prevent colonization by insects prior to their arrival to the study site and transported there in less than 2 h. The study site was a large abandoned agricultural field located approximately 40 km northeast of Moncton in Cocagne, New Brunswick, Canada (46°20.364′ N, 64°39.724′ W). Abundant vegetation in the study field included Canada goldenrod (*Solidago canadensis* L.), dandelion (*Taraxacum officinale* Weber ex Wiggers), flat-top white aster (*Doellingeria umbellata* (Mill.) Nees), grasses from the Poaceae family (e.g., *Festuca trachyphylla* (Hack.) Krajina, *Phleum pratense* L.), and red clover (*Trifolium pratense* L.). A few spaced trees were present within the field, such as tamarack (*Larix laricina* (Du Roi) K.Koch), grey alder (*Alnus incana* (L.) Moench ssp. *rugosa* (Du Roi) R.T.Clausen), black spruce (*Picea mariana* (Mill.) Britton, Sterns & Poggenb.), dogwoods (*Cornus spp.*), and spruces (*Picea spp.*).

On 6 July 2020, two fresh carcasses and two small bait traps were set at the study site in direct sunlight. These four experimental units were placed in a way to maximize the distance which separated them (Figure S1). The carcasses were positioned 144 m away from one another, the traps were positioned 149 m from one another and at least 90 m from carcasses. A distance exceeding 50 m prevents cross-contamination by crawling fly larvae between carcasses [25,26]. This trial ended on 20 July 2020 and the carcasses, which had all reached the dry decay stage, were removed. A new set of four experimental units (i.e., two new carcasses and two traps) were set at the study site on 5 August 2020 (Figure S1). These carcasses were 142 m away from one another and the traps were 147 m from one another and at least 98 m from carcasses. This second trial ended on 19 August 2020, when the carcasses had all reached the dry decay stage. 

The carcasses were placed on a 61 × 122 × 1 cm perforated plastic surface covered in soilless potting soil. The plastic surface allowed decomposition fluids and rain to leach away, delineated the sampling area, and facilitated the identification and recovery of insects that burrow in the soil or hide in the vegetation [27]. Each carcass was protected from vertebrate scavengers by a 61 × 61 × 122 cm cage made with chicken wire and anchored to the ground using pegs, bungee cords, and cinder blocks. Small bait traps consisted of “Flies Be Gone” commercial traps, modified to recover necrophagous Diptera. The closed bottom part of the trap was cut to create an opening and insert a 120 mL cylindrical plastic cup containing 60 g of minced pork liver as bait that was attached using duct tape. To hinder the direct colonization of the liver while allowing for the propagation of volatile organic compounds, the open end of the cup was wrapped in tulle fabric. Previously frozen baits were removed from the freezer and allowed to defrost and decompose at room temperature 24 h before the traps were set. When set, the traps were suspended from metal rods on-site, about 1.5 m from the ground and secured to the rod using zip ties.

### 2.2. Sampling Procedure

The sampling protocol was adapted from a previously published procedure [28]. During the decomposition process, the fauna visiting the carcasses was documented daily between 10:00 A.M. and 11:59 A.M. Carcasses were sampled in a random order. They were approached carefully to diminish disturbance of insects due to visitation. For up to 10 min, we observed the carcasses to tally the number of specimens per family. To complement this, a 10-min period was allocated for collection of flying insects using an entomological net. The few crawling insects that could not be identified in the field were collected using forceps for a maximum of 10 min. To minimize the impact of investigator disturbance on the decomposition process, less than 10% of any taxa was collected. The exact number of specimens collected was based on the number from each taxon that were visually tallied above. As can be seen in Section 3, only two families (i.e., Calliphoridae and Piophilidae) were abundant enough to be sampled from carcasses. Collected specimens were then placed in a jar containing 70% ethanol for preservation until further identification. Every three days, the small bait traps were replaced with new ones. This interval was used because in our geographic area, small bait traps are usually still empty after a single day of sampling.

All Calliphoridae and Piophilidae specimens were identified at the species level using Marshall et al. [29] and the Université de Moncton reference collection, with the exception of *Pollenia sp*. and *Protocalliphora sp.*, which were identified at the genus level. Other Diptera that were not abundant enough to be sampled from carcasses were identified at the family level. Specimens were deposited in the Université de Moncton reference collection. Air temperature was obtained from a federal weather station located in Bouctouche, approximately 17 km from the study site. The average temperature and postmortem accumulation of degree-days over 5 °C (ADD_5_) was calculated daily using the following formula: ADD_5_ = [{T_min_ + T_max_}/2] − 5 °C, where T_min_ and T_max_ represent the daily minimum and maximum air temperatures, respectively, and 5 °C represents the minimum developmental threshold over which the accumulation was considered. The 5 °C threshold was selected because it led to the best models in terms of overall fit and percentage of variability explained by these models.

### 2.3. Statistical Analyses

To allow for the comparisons between traps and carcasses, the Diptera documented on each carcass as well as the postmortem ADD_5_ during the trap sampling interval were individually summed up and the mean daily temperature was averaged for the same period. To examine the effects of postmortem ADD_5_ on the abundance of Calliphoridae and other dipterans on carcasses and in small bait traps, we used generalized additive mixed models (GAMMs). The abundance of observed Diptera identified to the family level was used for this analysis. The GAMMs accounted for the autoregressive structure of the data, the repeated measures and the Poisson data distribution [30]. To examine the effects of the carcasses on small bait traps and vice-versa, a GAMM including the abundance on the other type of unit as a covariate was carried out. The Sørensen’s and Percent similarity indices were calculated by comparing the species composition of adjacent pairs of carcasses and traps obtained from a combination of the abundance data from visual estimations and from the collection and subsequent identification of Diptera. A descriptive table presenting this data in terms of the percentage of the species encountered was also produced. The effects of postmortem ADD_5_ on the index values were examined using the same GAMMs as above but with Gaussian (Sørensen) and quasipoisson (Percent similarity) data distribution. The Sørensen’s and Percent similarity indices were also calculated by comparing species composition on carcasses exposed in the same month. Finally, we performed a canonical correlation analysis on data obtained from a combination of the abundance from visual estimations and from the collection and subsequent identification of Diptera using the *CCorA* function from the package *vegan*. Canonical correlation analysis allows an evaluation and an illustration of the relationships between two multivariate sets of variables, namely the study parameters (matrix of independent variables) and the abundance of each of the taxa (matrix of dependent variables). All statistical analyses were performed using R version 3.5.2 [31]. We ensured that all assumptions of each analysis were satisfied.

## 3. Results

### 3.1. Abundance Data

**Figure 1:**
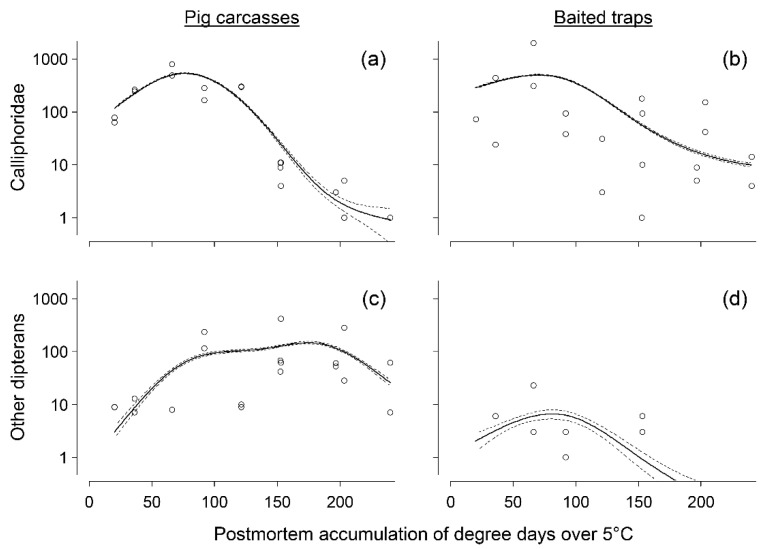
Dipterans documented as a function of postmortem accumulated degree-days over 5 °C, for: (**a**) Calliphoridae observed on carcasses (*r*^2^ = 0.63; *edf* = 3.903; *F* = 442; *p* < 0.01), (**b**) Calliphoridae captured in baited traps (*r*^2^ = 0.09; *edf* = 2.997; *F* = 676.7; *p* < 0.01), (**c**) other dipterans observed on carcasses (*r*^2^ = 0.05; *edf* = 3.961; *F* = 87.03; *p* < 0.01) and (**d**) other dipterans captured in baited traps (*r*^2^ = 0.10; *edf* = 2.555; *F* = 8.87; *p* < 0.01). The solid black line indicates the model prediction and the confidence interval of the prediction is illustrated with small dashes.

During the study, a total of 5001 Diptera specimens were captured using small bait traps and 4619 Diptera specimens were documented on the carcasses. Of the Diptera specimens collected in the traps, 98.0% were Calliphoridae, whereas they represented 40.3% of the specimens retrieved from the carcasses. Other Diptera represented 2.0% and 59.7% of the specimens in the baited traps and on the carcasses, respectively. For Calliphoridae, the abundance on the carcasses and in the baited traps reached a peak between 50 and 100 postmortem ADD_5_ before plummeting (Figure 1a,b). The relationship between the Calliphoridae observed on the carcasses and postmortem ADD_5_ was strong (i.e., *r*^2^ = 0.63) but was comparatively weak for those captured by the baited traps (i.e., *r*^2^ = 0.09) (Figure 1a,b). In fact, the abundance of Calliphoridae on the carcasses strongly influenced the abundance of Calliphoridae captured in the baited traps, with this being a positive relation (*F* = 21.602; *p* < 0.01). However, the presence of the traps did not have an effect on the abundance of Calliphoridae observed on the carcasses (*F* = 1.424; *p* = 0.22). Similarly, the abundance of other dipterans peaked between 50 and 200 postmortem ADD_5_ on carcasses and between 50 and 100 postmortem ADD_5_ in traps (Figure 1c,d). However, for both types of experimental units, the relationship between dipteran abundance and postmortem ADD_5_ was weak (i.e., *r*^2^ ≤ 0.10).

### 3.2. Comparison of Species Composition

**Figure 2:**
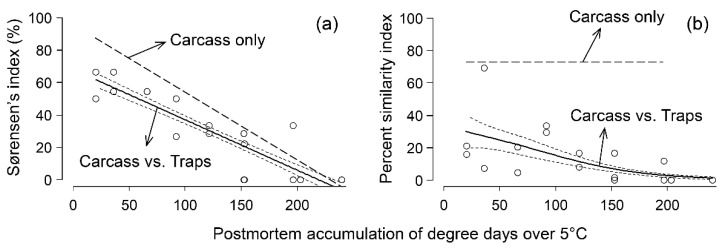
Comparison of Diptera documented in traps and on carcasses using the (**a**) Sorensen’s index (*r*^2^ = 0.78; *edf* = 1; *F* = 85.42; *p* < 0.01) and the (**b**) percent similarity index (*r*^2^ = 0.29; *edf* = 1.584; *F* = 14.72; *p* < 0.01) as a function of postmortem accumulated degree-days over 5 °C. The solid black line indicates the model prediction and the confidence interval of the prediction is illustrated with small dashes. For comparison purposes, the large dash line indicates the value of the index when used for carcass to carcass comparison.

The assemblages of Diptera documented on carcasses and in traps are presented in Table 1. The most abundant Calliphoridae associated with the carcasses were *Phormia regina* (25.6%), *Lucilia illustris* (9.0%), and *Pollenia sp.* (3.2%) (Table 1). Piophilidae (i.e., all *Stearibia nigriceps*: 48.6%), Sepsidae (4.1%), Muscidae (4.9%), Drosophilidae (0.9%), Sarcophagidae (0.8%), Anthomyiidae (0.4%), and Phoridae (0.1%) specimens were also sampled from the carcasses (Table 1). In the traps, the most commonly recovered Diptera were Calliphoridae, of which *L. illustris* (72.1%), *P. regina* (21.0%), and *Pollenia sp.* (4.1%) were most abundant (Table 1). Muscidae (0.8%), Sarcophagidae (0.5%), and Anthomyiidae (0.7%) were also recovered from the traps (Table 1). Five species captured by the traps were not sampled on the carcasses (Table 1), whereas one Calliphoridae genus was sampled only on the carcasses (*Protocalliphora sp.*). Drosophilidae, Sepsidae, Phoridae, and Piophilidae were never captured by the baited traps despite their presence on the carcasses (Table 1). When assemblages in traps and on carcasses were compared in terms of Diptera occurrence (Sørensen’s index, Figure 2a) and abundance (Percent similarity index, Figure 2b), the highest similarity between carcasses and traps was observed below 50 postmortem ADD_5_. Sørensen’s index indicated that a maximum of 60% of species or families were shared between traps and carcasses early in the decomposition process (Figure 2a). In contrast, the percent similarity index indicated that the level of similarity in species composition documented within traps and on carcasses in the same period was 30% at best (Figure 2b). Afterward, the Diptera assemblages increasingly differed between carcasses and traps (Figure 2a,b). By comparison, 90% of the species that were present early in decomposition were shared between pairs of carcasses, after which the level of similarity decreased steadily (Figure 2a). The species composition of necrophagous Diptera assemblages on the carcasses were also highly similar throughout decomposition (~75% similarity) until no insects were observed (i.e., postmortem ADD_5_ > 200; Figure 2b). 

**Figure 3:**
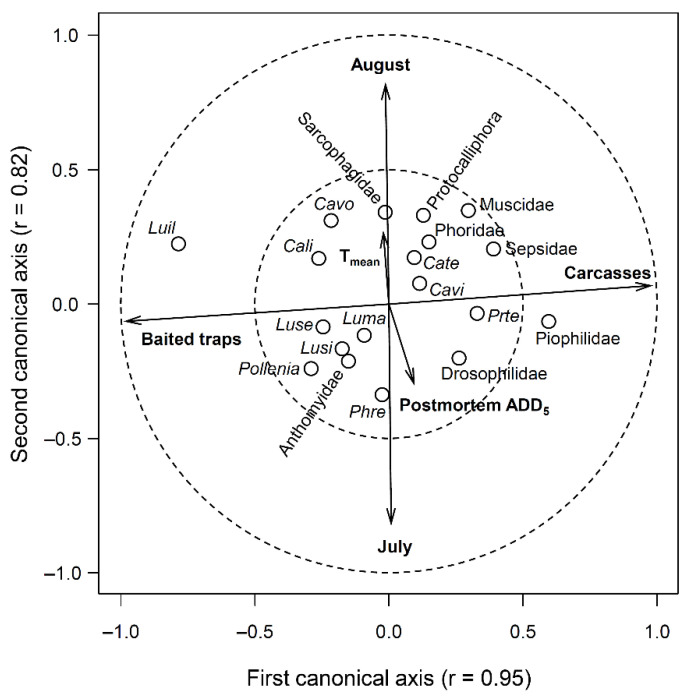
Canonical correlation analysis (Pillai’s trace = 2.68; adjusted redundancy *r*^2^ = 0.37; *p* < 0.01) between Diptera abundance (empty circles) and the study variables (i.e., type of experimental unit, sampling months, postmortem accumulated degree-days over 5 °C, and mean temperature—T_mean_; arrows). Calliphoridae species are identified using the acronym of the first two letters of their genus and species.

To identify the taxa responsible for these differences, we produced a canonical correlation (Figure 3). The first canonical axis separated the Diptera assemblages from carcasses and traps (Figure 3). All other explanatory variables (i.e., postmortem ADD_5_, mean temperature, sampling month) loaded onto the second canonical axis, indicating that the effect of the type of experimental unit is largely independent of them (Figure 3). *Lucilia illustris* was largely overrepresented by the traps and *Calliphora vomitoria*, *C. livida*, *L. sericata*, *L. silvarum*, *L. magnicornis*, *Pollenia sp.*, and Anthomyiidae were also recovered more frequently in traps than on carcasses (Table 1; Figure 3). Conversely, *Protocalliphora sp*., *C. terraenovae*, *C. vicina*, *Protophormia terraenovae*, Muscidae, Phoridae, and Drosophilidae were more abundant on the carcasses than in the traps (Table 1; Figure 3). Muscidae, which on average represented 4.9% and 13.4% of the dipterans found on the carcasses throughout the decomposition and in early decomposition respectively, represented only 0.8% of trapped dipterans (Table 1). Similarly, *C. terraenovae*, *C. vicina*, Sarcophagidae, and Anthomyiidae were common on carcasses (composition > 1%) early in decomposition (i.e., PMI 0‒2) but were rarely retrieved from traps (composition < 1%) (Table 1). *Phormia regina* and Sarcophagidae were equally abundant in the traps and on the carcasses (Table 1; Figure 3). However, Sarcophagidae represented a greater proportion of the specimens recovered from the carcasses in early decomposition than was represented by the traps (Table 1).

## 4. Discussion

Small bait traps are useful in forensic entomology to maintain a database of local and seasonal forensically important necrophagous insects [20,32,33,34], which may be crucial for analysis of entomological evidence in a given area [20]. That being said, the correlation between assemblages of Diptera captured by small bait traps and found on cadavers/carcasses has never been studied simultaneously, although some authors have used pre-existing data or literature to compare data [1,20,21]. However, these methods may be problematic because geography, climate, species-specific habitat associations, and seasons are known to have an important impact on Diptera communities [11,19,35,36,37,38]. Additionally, some authors have demonstrated the importance of biases associated with small bait traps, such as those caused by weather factors, height of traps, and position, as well as biased sex ratios and age-classes of captured Diptera [11,39,40]. Furthermore, the flight patterns of Calliphoridae are known to vary throughout the day [41,42]. As such, from an experimental standpoint, small bait traps which sample constantly are more likely to capture taxa which may not be active when the carcasses are being sampled. The aforementioned factors which influence Diptera assemblages and the many possible biases made us question the reliability of the previous comparisons between carcasses/cadavers and traps. These doubts have arisen because the experimental units were often geographically distant, located within different environments, and exposed to different climates and seasons. 

The present study compared carcasses and small bait traps exposed to the same microclimate and community while avoiding cross-contamination by crawling insects. We documented a similar abundance of Diptera in early decomposition between traps and carcasses. However, the composition and richness of the Diptera assemblages found in the small bait traps and on the carcasses differed significantly even in early decomposition, while these were very similar when comparing only the carcasses. These results support our predictions and the hypothesis that the attractiveness of decomposing animal matter to Diptera changes with species interactions, decomposition, and amount of available tissue. Despite previous studies claiming the opposite [1,20], we found little evidence to support the idea of a resemblance between the Diptera assemblages during early decomposition on the carcasses and in small bait traps, at least in our study area. In addition, the data did not support the idea that the assemblages within small bait traps may be representative of a later stage of decomposition.

### 4.1. Discrepancies between Small Bait Traps and Remains

Our analysis indicated that the differences in species compositions were mainly caused by the overrepresentation of *L. illustris* in trap captures. This may be caused, in part, by the inability of this species to compete with the dominant species on the carcasses, such as *P. regina* [43]. Other Calliphoridae species such as *C. vomitoria* and *L. sericata* were also overrepresented in trap captures. This was expected because certain necrophagous insects are known to be more drawn to smaller baits [5], and because previous studies have suggested that the Diptera collected from baited traps may provide an exaggerated representation of those on cadavers [33]. Furthermore, studies have shown that certain Diptera species preferentially colonize carrion at greater heights from ground level than others [44]. Comparatively, other Diptera such as *Protocalliphora sp.* and *C. terraenovae* were sampled in greater abundance from the carcasses than by the traps, in agreement with field observations that many necrophagous Diptera species are probably more attracted to larger amounts of animal carrion [5,15,21,22]. Furthermore, a study in Brazil which compared the Diptera associated with 14 murder cases and traps set at the scene of the death found that many species were present in the traps while they were absent from the cadavers [33]. Similarly, recent work, which compared the initial species composition of piglet carcasses and baited beef liver traps placed in the same habitat but during subsequent years, demonstrated that the three dominant Calliphoridae species found on the carcasses did not all correspond to those captured by the baited traps [20]. However, unlike Weidner et al. [20], many of the early-colonizing Diptera retrieved in this study from the carcasses during early decomposition (i.e., PMI 0–2) were rarely recovered from the traps (e.g., *C. terraenovae*).

Diptera other than Calliphoridae were also underrepresented in trap captures. For example, the total proportion of Muscidae captured by the small bait traps was lower than that found on the carcasses by a ratio of 5:1. This may be related to the presence of excrement released by the carcasses [45,46] but further studies will be needed to test this hypothesis. We also suspect that the absence of smaller Diptera (i.e., Piophilidae, Sepsidae, and Phoridae) in the traps could be due, in part, to trap design but further work will be needed to confirm this. Trapping studies using small bait traps rarely document smaller Diptera, although Schoenly traps, Malaise traps, yellow pan traps, or sweeping over a bait have been successful in capturing Piophilidae and Sepsidae [47,48]. It is important to mention that previous studies of small bait traps only reported Calliphoridae, even though the other families included in our study are of forensic relevance. Nevertheless, had we done the same, our conclusions would not have changed because the Calliphoridae complex is largely responsible for the differences documented here.

### 4.2. Other Considerations

The potential reasons for the sampling differences of the Diptera species and families documented are numerous. First, the fact that our bait was frozen and thawed may be involved, but this remains questionable. While one study reported that frozen meat attracts Calliphoridae differently than never-frozen meat [49], another study found no difference in Calliphoridae activity between refrigerated and frozen pig carcasses [50]. Second, the two sampling methods greatly differed, as the traps are passive, and the use of a sweep net is more active. For this reason, it is more likely for the data retrieved from sweeping over the carcasses to be biased, depending on the individual carrying out the sampling procedure. However, sweep net sampling remains the most widely used method for retrieving flying insects from a corpse during forensic investigations. For this reason, despite the biases this methodology may introduce, it was necessary in this study to allow for a proper comparison.

An interesting aspect of the current study is that trap captures are affected by the proximity of decomposing animal matter. This source of bias should be considered when setting small bait traps for necrophagous Diptera in an urban setting where trashcans containing decomposing animal tissues (e.g., meat) may be nearby. Moreover, the influence of carcasses on trap catches suggests that without the presence of the carcasses to attract necrophagous insects from afar, the insect assemblages captured by the small bait traps could have been even less similar to those found on the carcasses than what were documented here. One way to test this would be to place traps at increasing distances from carcasses in a given habitat.

Finally, one of the singular aspects of this study is the statistical effect size (i.e., the magnitude of the experimental effect [31]), which tells us a lot about the disparity between what is sampled by small bait traps and on carcasses. In the present study, the effect size was so large that after performing a first fully replicated experiment in July, the results were already clear and very significant. We did however replicate the experiment a second time in August to ensure that these results were not obtained by chance and documented the same consistent differences. Thus, despite the small sample size, the effect size was so large with eight experimental units (and 9620 documented Diptera) that we did not have any reason to believe that additional experimental units were needed.

## 5. Conclusions

While the results of the current study are in contradiction to the claim that small bait traps could be an accurate predictor of early arriving Diptera of forensic importance [1], they are not surprising. As early as the 1940s, a descriptive study carried out in South Africa had reached the same conclusions [51]. In addition, the effects of abundance and type of necromass on colonizing insect communities have been documented beyond doubt on other decaying substrates (e.g., dung, dead wood: [52,53,54,55]). Moreover, in previous study of sapromyiophilous plants that attract fly pollinators by mimicking carrion and dung odors, antennae of houseflies showed positive dose-dependent responses to the volatile compounds released by flowers [56]. There is no rationale why these effects should be any different with carrion. It appears implausible that a time-limited sampling protocol could be comparable to method sampling around the clock. In addition, considering that traps are often baited with homogeneous tissues from internal organs, there is a strong possibility that they emit different volatile organic compounds than whole carcasses [57]. Realistically, the effect of the bait itself in small bait traps still needs to be tested using proper design, solid controls and appropriate statistics [31]. To verify the untested assumption discussed above that bait emits volatile organic compounds similar to those emitted by a cadaver/carcass, the first logical step would be to perform a chemical analysis of the volatile organic compounds released by different types of bait and carcasses or cadavers throughout decomposition. This leads us to suggest that although our results are not necessarily applicable to all trap designs or locations, if the trap in question is baited with a small amount of animal tissue, there is no reason to believe that the results will be different. In the meantime, small bait traps should be considered useful tools to provide baseline data on Diptera occurrence if cadavers or large carcasses are not readily available. In agreement with Arnaldos et al. [58], we stress that caution must be exercised when extrapolating data obtained from non-human experimental methods to forensic cases, and especially when using data from small bait traps in courtroom proceedings.

## Figures and Tables

**Table 1:** Percentage of specimens of each species, genera and family of Diptera recovered during successive sampling intervals (PMI) from carcasses (*n* = 4) and small traps baited with pork liver (*n* = 4) in Cocagne, New Brunswick in July and August 2020.

Experimental Unit	Carcasses												Traps											
Sampling Month	July					August					Average		July					August					Average	
Postmortem Interval (PMI)	0–2	3–5	6–8	9–11	12–14	0–2	3–5	6–8	9–11	12–14	0–2	0–14	0–2	3–5	6–8	9–11	12–14	0–2	3–5	6–8	9–11	12–14	0–2	0–14
*Lucilia illustris* (Meigen)	35.7	13.1	‒	5.0	‒	36.0	‒	‒	‒	‒	35.9	9.0	87.7	42.4	74.3	75.7	57.1	89.8	52.9	57.8	94.3	88.9	88.8	72.1
*Phormia regina* (Meigen)	14.3	81.0	84.6	10.0	7.1	22.0	34.8	2.4	‒	‒	18.1	25.6	1.4	54.2	14.3	16.4	42.9	5.9	37.0	28.4	4.2	5.6	3.7	21.0
*Pollenia sp.* Robineau-Desvoidy	14.3	‒	2.6	5.0	7.1	2.0	1.1	‒	‒	‒	8.1	3.2	11.0	2.0	5.7	7.3	‒	2.5	2.2	3.4	1.0	5.6	6.8	4.1
*Protophormia terraenovae* (Robineau-Desvoidy)	‒	2.4	5.1	‒	‒	2.0	2.2	‒	‒	‒	1.0	1.2	‒	0.2	‒	‒	‒	‒	‒	‒	‒	‒	‒	0.0
*Calliphora terraenovae* Macquart	7.1	‒	‒	‒	‒	‒	‒	‒	‒	‒	3.6	0.7	‒	‒	‒	‒	‒	‒	1.4	‒	‒	‒	‒	0.1
*Calliphora vicina* Robineau-Desvoidy	3.6	‒	‒	‒	‒	‒	‒	‒	‒	‒	1.8	0.4	‒	0.0	‒	‒	‒	‒	‒	‒	‒	‒	‒	0.0
*Lucilia sericata* (Meigen)	‒	‒	‒	‒	‒	‒	‒	‒	‒	‒	‒	‒	‒	‒	2.9	‒	‒	‒	0.7	‒	‒	‒	‒	0.4
*Calliphora livida* Hall	‒	‒	‒	‒	‒	‒	‒	‒	‒	‒	‒	‒	‒	0.0	‒	‒	‒	‒	1.4	‒	0.5	‒	‒	0.2
*Protocalliphora sp.* Hough	‒	‒	‒	‒	‒	2.0	‒	‒	‒	‒	1.0	0.2	‒	‒	‒	‒	‒	‒	‒	‒	‒	‒	‒	‒
*Lucilia silvarum* (Meigen)	‒	‒	‒	‒	‒	‒	‒	‒	‒	‒	‒	‒	‒	‒	‒	0.6	‒	‒	‒	‒	‒	‒	‒	0.1
*Calliphora vomitoria* (Linnaeus)	‒	‒	‒	‒	‒	‒	‒	‒	‒	‒	‒	‒	‒	‒	‒	‒	‒	0.2	‒	‒	‒	‒	0.1	0.0
*Lucilia magnicornis* (Siebke)	‒	‒	‒	‒	‒	‒	‒	‒	‒	‒	‒	‒	‒	0.0	‒	‒	‒	‒	‒	‒	‒	‒	‒	0.0
Piophilidae—*Stearibia nigriceps* (Meigen)	3.6	3.6	‒	60.0	78.6	14.0	33.7	92.9	100.0	100.0	8.8	48.6	‒	‒	‒	‒	‒	‒	‒	‒	‒	‒	‒	‒
Muscidae	10.7	‒	2.6	5.0	7.1	16.0	7.9	‒	‒	‒	13.4	4.9	‒	1.1	‒	‒	‒	0.8	2.2	4.3	‒	‒	0.4	0.8
Sepsidae	3.6	‒	2.6	10.0	‒	2.0	18.0	4.8	‒	‒	2.8	4.1	‒	‒	‒	‒	‒	‒	‒	‒	‒	‒	‒	‒
Sarcophagidae	3.6	‒	‒	‒	‒	4.0	‒	‒	‒	‒	3.8	0.8	‒	0.0	‒	‒	‒	0.4	0.7	3.4	‒	‒	0.2	0.5
Anthomyiidae	3.6	‒	‒	‒	‒	‒	‒	‒	‒	‒	1.8	0.4	‒	‒	2.9	‒	‒	‒	1.4	2.6	‒	‒	‒	0.7
Drosophilidae	‒	‒	2.6	5.0	‒	‒	1.1	‒	‒	‒	‒	0.9	‒	‒	‒	‒	‒	0.2	‒	‒	‒	‒	0.1	0.0
Phoridae	‒	‒	‒	‒	‒	‒	1.1	‒	‒	‒	‒	0.1	‒	‒	‒	‒	‒	‒	‒	‒	‒	‒	‒	‒

## Data Availability

The data presented in this study are available on request from the corresponding author. The data are not publicly available due to unfinished related ongoing further studies and manuscript.

## Supplementary Materials

The following are available online at https://www.mdpi.com/2075-4450/12/3/261/s1. Figure S1: Map of the study site in Cocagne, New Brunswick, Canada, with location of pigs (squares) and small bait traps (circles) in the July and August trials.
