# Supplementary material for: Small Bait Traps May Not Accurately Reflect the Composition of Necrophagous Diptera Associated to Remains
Source: Insects. 2021 Mar 20;12(3):261. doi: 10.3390/insects12030261 (PMC8003588; doi:10.3390/insects12030261)
Supplement: Supplementary file 1 [file insects-12-00261-s001.pdf]

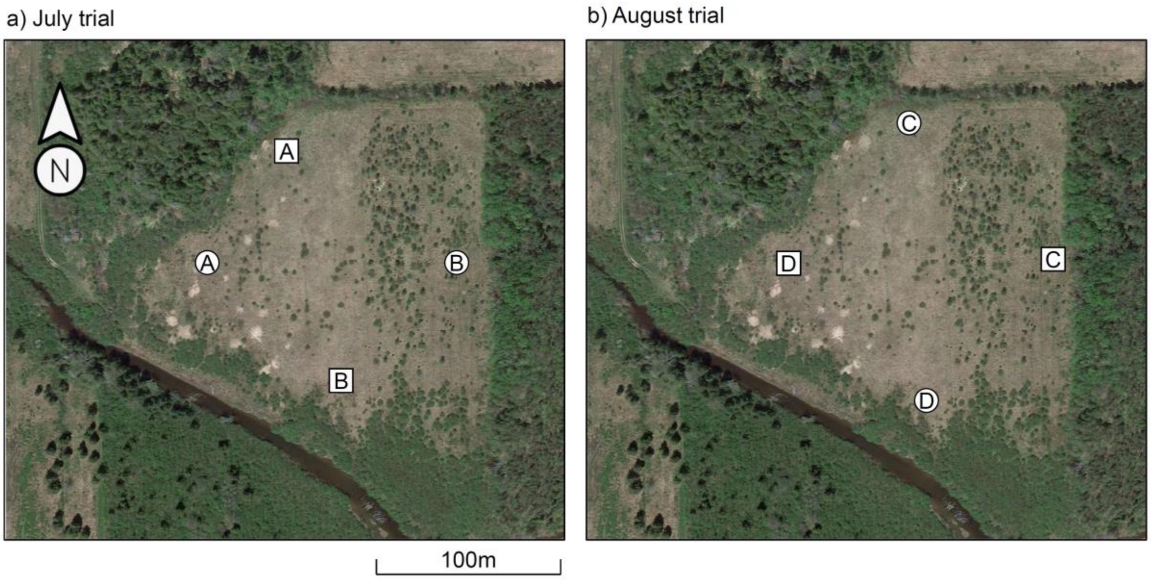

Figure S1. Map of the study site in Cocagne, New Brunswick, Canada, with location of pigs (squares) and small bait traps (circles) in the July and August trials.
